# Emergency Department Reconsultations After a Secondary Prevention Bundle for Medication-Related Problems: A Retrospective Cohort Study

**DOI:** 10.3390/jcm14196907

**Published:** 2025-09-29

**Authors:** Adrián Plaza-Díaz, Ana Juanes-Borrego, Natalia Sanz-Lopez, Javier González-Bueno, Jordi Fernández-Morató, Milagros García-Peláez, Jesús Ruiz-Ramos

**Affiliations:** 1 Pharmacy Department, Hospital de la Santa Creu i Sant Pau, Sant Antoni Maria Claret 167, 08025 Barcelona, Spain; ajuanes@santpau.cat (A.J.-B.); jrzrms@gmail.com (J.R.-R.); 2 Institut de Recerca Sant Pau (IR SANT PAU), Sat Quintí 77-79, 08041 Barcelona, Spain; 3 Department of Medicine, Universitat Autònoma de Barcelona, 08193 Bellaterra, Spain; 4 Emergency Department, Hospital de la Santa Creu i Sant Pau, Sant Antoni Maria Claret 167, 08025 Barcelona, Spain; nsanzl@santpau.cat; 5 Pharmacy Department, Hospital Dos de Maig Consorci Sanitari Integral, 08025 Barcelona, Spain; jgonzalezb@csi.cat; 6 Central Catalonia Chronicity Research Group (C3RG), University of Vic—Central University of Catalonia (UVIC-UCC), 08500 Vic, Spain; 7 Pharmacy Department, Consorci Sanitari de Terrassa, 08227 Terrassa, Spain; jfernandezm@cst.cat; 8 Pharmacy Department, Hospital General de Granollers, 08402 Granollers, Spain; icosgp@gmail.com

**Keywords:** drug-related problems, emergency department, secondary prevention, readmission, older adults

## Abstract

**Background/Objective:** Drug-related problems (DRPs) are a common, potentially avoidable cause of emergency department (ED) use. In December 2022, our hospital integrated a pharmacist-led intervention into routine ED practice. This intervention comprised medication optimization, adherence counseling, and coordinated hand-off to primary care. We quantified 30- and 90-day reconsultations after discharge and explored factors associated with DRP-related revisits. **Methods**: A retrospective cohort of adults (≥18 years) who attended a tertiary ED (Barcelona, Spain). We included index DRP visits from 1 December 2022 to 30 June 2024. All received the bundle. Demographic, clinical, and pharmacotherapeutic data were extracted from the Catalan Shared Health Record; an independent committee classified revisits as a DRP or non-DRP. Predictors of 30-day DRP revisits were assessed with multivariable logistic regression. **Results**: Among 1247 patients (mean age 78.6 ± 16.2 years; 59.2% women; and median nine drugs), 120 (9.6%) reconsulted the ED within 30 days, and 194 (15.5%) within 90 days for any cause. DRP-specific rates were 30.8% (37/120) at 30 days and 26.3% (51/194) at 90 days; 81% and 80% of these revisits, respectively, involved a recurrence of the same DRP. The most frequent index DRPs were constipation (14.2%), gastrointestinal bleeding (9.2%), hypertension (8.3%), seizures (8.3%) and hyponatraemia (6.7%). An age ≥ 80 years independently predicted fewer 30-day DRP revisits (OR 0.32; 95% CI 0.13–0.79); hypertension and cognitive impairment were not significant after adjustment. **Conclusions**: In this single-arm implementation cohort, overall, 30-day ED reconsultations were 9.6% and about one-third were DRP-related, predominantly recurrences, and chiefly gastrointestinal bleeding and seizures. These descriptive findings should be interpreted cautiously given potential survivorship bias and residual confounding; the apparently lower risk among patients aged ≥ 80 years is hypothesis-generating and may reflect geriatric care pathways and caregiver engagement. Targeted post-discharge monitoring for high-recurrence DRPs may help reduce avoidable ED use, and future evaluations should test this in quasi-experimental or randomized designs.

## 1. Introduction

The progressive aging of the population and the increase in healthcare needs worldwide have led to a sustained rise in the demand for medical care, especially in the emergency department (ED). This phenomenon is particularly observed among vulnerable individuals with chronic diseases, multimorbidity, polypharmacy, dependence, and other long-term conditions, who are at a higher risk of experiencing drug-related problems (DRPs) that require medical attention [1,2,3,4].

DRPs, defined as health issues that patients experience due to the use, misuse, or omission of medications (including adverse drug reactions, medication errors, interactions, and adherence problems), represent a significant public health issue. It is estimated that they account for between 7.4% and 37.6% of ED consultations, with most of them being preventable [5,6,7]. Additionally, DRPs are responsible for approximately 5% to 10% of hospitalizations and up to 21% of readmissions, many of which could be prevented [8,9,10,11]. Around 33% of patients with DRPs have a return visit to the ED within 30 days of discharge [12].

Several studies have reported a reduction in ED revisits and hospital readmissions after the implementation of secondary prevention programs in those patients with DRPs. “Medication Code,” a program consisting of applying a secondary prevention bundle for DRPs in ED consultations (which includes the optimization of chronic prescription, improving therapeutic adherence, and strengthening care coordination), has been shown to reduce the 30-day hospital readmission rate for any cause and to be cost-efficient [13,14].

However, there is limited information regarding the pattern of revisits when the program is routinely and sustainably integrated into a high-complexity ED. This study describes the frequency, characteristics, and factors associated with reconsultations within 30 days for another DRP after 18 months of continuous implementation of the “Medication Code” bundle in routine clinical practice. Pre-implementation, our ED’s most common DRP presentations were antithrombotic-related bleeding, central nervous system drug-related falls, and constipation due to high anticholinergic burden, consistent with Spanish multicenter and single-center ED studies [5,6].

To distinguish it from prior ED pharmacist services, the “Medication Code” was designed as a standardized, single-episode bundle embedded within the ED workflow. It mandates a 48 h pharmacist follow-up call, transmits a structured hand-off to primary care through the Catalan Shared Health Record, and leverages the EFAD clinical–pharmacy network across care levels

The aim of this study was to describe 30-day reconsultations after discharge following the implementation of a secondary prevention bundle for DRPs in routine clinical practice among patients who visited the ED due to a medication-related condition (including adverse drug reactions, dosing errors, interactions, and clinically relevant non-adherence).

The secondary objectives were to describe the characteristics of patients who revisited the ED within 90 days after discharge and to assess differences between patients with new ED consultations related and unrelated to a subsequent DRP episode.

## 2. Materials and Methods

### 2.1. Study Design

We conducted a single-arm retrospective implementation cohort. The study took place at Hospital de la Santa Creu i Sant Pau (HSCSP) in Barcelona, a university-affiliated tertiary hospital that serves a catchment of approximately 407,000 residents and manages around 150,000 ED visits each year. In Catalonia, citizens are assigned to health regions based on postal address; HSCSP is the referral center for the AIS Barcelona Dreta region. The EFAD (Equip de Farmàcia Assistencial de Barcelona Dreta) network comprises clinical pharmacists who collaborate with physicians and nurses across care levels within this region. The Catalan public health system operates a territory-wide shared electronic health record, allowing authorized providers to access patients’ longitudinal medical information throughout the network. Eligible participants were adults (≥18 years) whose index ED visit was adjudicated as due to a drug-related problem (DRP) between 1 December 2022 and 30 June 2024 and treated with the secondary prevention bundle, “Medication Code”, which was the standard of care and was applied to **all** eligible DRP patients before ED discharge; no discretionary selection was used.

The Medication Code program included activities aimed at optimizing patients’ chronic prescriptions (through patient interviews and reviews of their chronic medication regimens), improving therapeutic adherence (including the delivery of written information about the medication treatment plan and a telephone consultation 48 h after discharge) and enhancing coordination across different healthcare levels (including sending an email to the next healthcare provider summarizing the reason for the consultation and any changes in the medication treatment) [13]. Exclusion criteria were patients with hospitalization longer than 30 days after the initial visit and death during the index episode.

At admission, we recorded selected variables that could modify the effects of DRPs according to the information obtained in previous studies [15]: age, sex, number of chronic conditions, number of medications being taken, hypertension, diabetes mellitus, dyslipidemia, ischemic heart disease, atrial fibrillation, heart failure, chronic kidney disease stage 3 or worse (estimated glomerular filtration rate < 60 mL/min/1.73 m^2^), active oncological disease, and cognitive impairment according to the Global Deterioration Scale of Reisberg [16]. This information was collected by a retrospective review of the Clinical Health Shared Record of Catalonia (CHSRC).

The main outcome variable was a dichotomous (yes/no) indicator of any unplanned ED reconsultation within 30 days after discharge from the index DRP visit. Planned/scheduled visits were excluded; if multiple ED contacts occurred within 30 days, only the first qualifying event was counted. The diagnosis for each 30-day reconsultation was classified as related to a DRP or to other causes according to the criteria of the multidisciplinary clinical committee, which reviews these cases as part of the quality assurance and continuous improvement policy of the Medication Code program.

A multidisciplinary committee (three clinical pharmacists, one ED physician) independently reviewed revisits using explicit criteria (temporal plausibility; pharmacology; dechallenge/rechallenge when available; and alternative explanations). Disagreements were resolved by consensus; members were not blinded to the index DRP or bundle exposure.

Cerebrovascular events were classified as DRP-related only when medication causality was plausible based on temporal sequence, pharmacologic mechanism, and documentation (e.g., anticoagulant-associated intracranial hemorrhage, drug–drug interaction, or documented non-adherence); otherwise, they were adjudicated as non-DRP revisits.

### 2.2. Intervention

A structured secondary prevention bundle for DRPs was applied once to every patient. A clinical pharmacist coordinated the bundle alongside two ED physicians and the Barcelona EFAD pharmacy network, working in partnership with the wider multidisciplinary team. Admission decisions in the ED remained entirely under the usual medical team.

#### 2.2.1. Optimizing Long-Term Medication

1a. Medication history.

The ED pharmacist conducted a face-to-face (or telephone) interview with the patient or caregiver, compiled the complete home medication list, and entered it into the Catalan Shared Health Record (HCCC), a region-wide electronic record visible to all providers and community pharmacies.

1b. Reconciliation at every care transition.

Following Institute for Healthcare Improvement guidance, the pharmacist compared the pre-admission list with prescriptions at admission, transfer, and discharge. Discrepancies were discussed with prescribers and corrected and documented in the electronic record for the next care level and the patient.

1c. Structured medication review.

Using the three-step method of Espaulella-Panicot et al. [17,18]

1. Patient-centered evaluation—determine the overarching care goal (survival, functional maintenance, or symptom control).
2. Diagnosis-centered review—match each health problem with its drug therapy, ensuring alignment with the agreed care goal.
3. Medication-centered assessment—balance benefits and risks of each drug in light of the patient’s clinical status.

#### 2.2.2. Enhancing Adherence

2a. Written medication plan.

At ED or ward discharge, patients received a clear written regimen incorporated into the electronic discharge summary and accessible to all providers.

2b. A 48 h follow-up call.

The pharmacist telephoned the patient, caregiver, or nursing-home staff two days post-discharge to confirm resolution of the initial problem and understanding of the treatment plan.

#### 2.2.3. Strengthening Care Coordination

A structured electronic report was sent to the primary care provider via the electronic communication system set up by the Catalan health system with the primary care pharmacist, who shared the information with the rest of the team (physicians and nurses), detailing the DRP that precipitated the ED visit, discharge medications, and medium- and long-term pharmacotherapy recommendations.

### 2.3. Statistical Analysis

Patients who died during hospitalization or those hospitalized for >30 days were excluded from the analysis because the main and secondary outcome variables could not be assessed.

Given the binary dependent variable, we used logistic regression to estimate odds ratios (ORs). ORs were calculated, adjusted analyses controlled for covariates that showed an association with hospital readmission or ED reconsultation at *p* < 0.100 in univariable screening, and we report adjusted odds ratios (ORs) with 95% confidence intervals (CIs). The multivariable model included pre-specified patient characteristics with established links to ED use/readmission: age ≥80 years, chronic heart failure, atrial fibrillation, chronic kidney disease, active malignancy, hypertension, diabetes mellitus, ischemic heart disease, and major polypharmacy (defined as >10 regular medications in the electronic prescription record).

The analyses were conducted using Stata BE version 17.0 (Stata-Corp LLC, College Station, TX, USA).

## 3. Results

During the study period, 1247 patients were included, all of whom received the “Medication Code” secondary prevention bundle. A total of 120 patients (9.6%) reconsulted the ED for any cause within 30 days. The main baseline characteristics are shown in Table 1.

### 3.1. Index and Recurrent DRP

**Figure 1:**
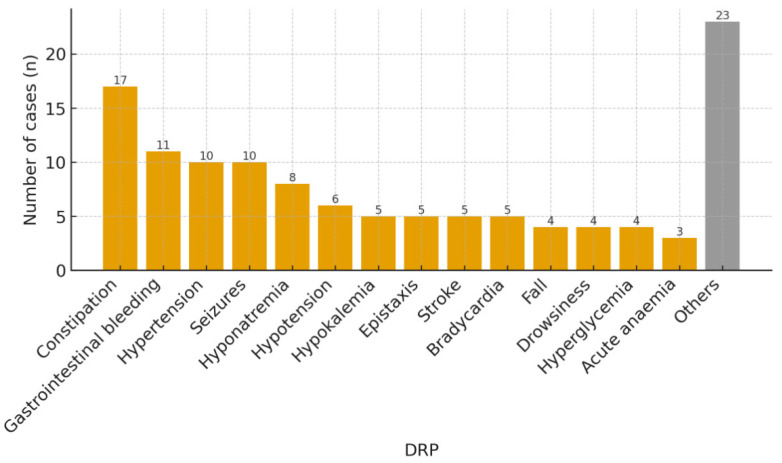
DRP that triggered activation of the secondary prevention bundle during the initial ED visit. Low-frequency categories (n < 3 per category) were grouped as “Others” to improve readability.

**Figure 2:**
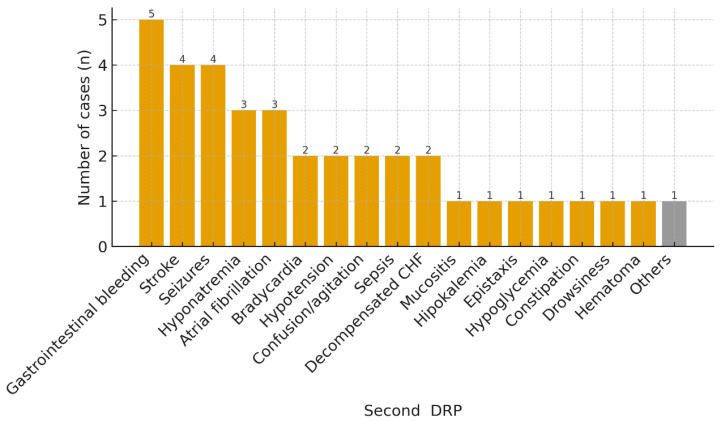
DRP prompting the return ED visit after implementation of the prevention bundle. Abbreviations: CHF, congestive heart failure.

The most common DRPs that motivated the activation of the secondary prevention bundle in these 120 patients were constipation (17, 14.2%), gastrointestinal bleeding (11, 9.2%), hypertension (10, 8.3%), seizures (10, 8.3%), and hyponatremia (8, 6.7%) (Figure 1). “Other” DRP groups were low-frequency categories with fewer than three cases each. Although “Other” DRPs constitute the largest volume, it is highly heterogeneous and was not analyzed in depth. A total of 37 patients (30.8%) reconsulted the ED for a new DRP, with the most frequent being gastrointestinal bleeding (5, 13.5%), stroke (4, 10.8%), seizures (4, 10.8%), and hyponatremia (3, 8.1%) (Figure 2); 30 of them (81.1%) revisited the ED for the same DRP as the primary one. The mean time until reconsultation was 24.6 days (SD ± 22.8). The ATC groups most commonly involved were C—cardiovascular system (35, 29.2%), B—blood and blood forming organs (26, 21.7%), N—nervous system (26, 21.7%), and A—alimentary tract and metabolism (23, 19.2%).

### 3.2. Predictors of 30-Day DRP Revisits

In the univariate analysis, the variables associated with 30-day readmission due to another DRP (*p* < 0.100) were as follows: age ≥ 80 years, hypertension, and cognitive impairment. The results of the multivariate analysis are shown in Table 2. An age of ≥80 years was associated with a lower probability of reconsultation due to a new DRP within 30 days (OR 0.32; 95% CI 0.13–0.79).

### 3.3. 90-Day ED Reconsultations and DRP Recurrence

Regarding the secondary objective, 194 patients (15.5%) reconsulted the ED for any cause after the implementation of the secondary prevention bundle 90 days after hospital discharge. The most common DRPs that motivated the activation of the secondary prevention bundle in these 194 patients were constipation (26, 13.4%), gastrointestinal bleeding (19, 9.8%), hypertension (16, 8.2%), hypotension (13, 6.7%), and seizures (11, 5.7%). A total of 51 patients (26.3%) reconsulted for another DRP, 41 of whom (80.4%) reconsulted for the same DRP.

## 4. Discussion

In this single-arm cohort, almost 1 in 10 patients reconsulted the ED within 30 days. Because patient populations, eligibility criteria, and outcome ascertainment differ from prior studies, including the randomized trial [13], direct rate comparisons are not appropriate; our estimate is an implementation metric rather than an effect size. The overall reconsultation rate was below 10%. Although numerically lower than in some reports [12,19], cross-setting benchmarking is limited by differences in health system structures, case mixes, and discharge pathways, and any apparent differences should be interpreted cautiously. Our findings should be interpreted as implementation results from a single center and not as evidence of effect. A more suitable approach to estimate effect under routine care would be a quasi-experimental or randomized design.

The finding that an age of ≥80 years acts as a protective factor against reconsultation due to a new DRP contrasts with the evidence for other clinical conditions (such as heart failure, infections, or fractures) in which advanced age is among the main risk factors for an early return to the ED [20,21,22]. Possible explanations include the preferential referral of octogenarians to short-stay geriatric units with protocolized follow-up or a higher degree of caregiver adherence to the pharmaceutical plan. A survival bias cannot be excluded, because patients with the worst prognoses (death during the index admission or within 30 days) were excluded by design. Importantly, in the multivariable model, an age of ≥80 years remained the only independent predictor of lower 30-day, DRP-related ED reconsultation after adjustment for recorded comorbidities; however, residual confounding by unmeasured domains (frailty, functional status, and social support) may partly explain this association. Taken together, these patterns are descriptive and should be interpreted cautiously given the potential for survivorship bias and residual confounding; accordingly, the apparent lower risk among ≥80-year-olds is hypothesis generating.

The high proportion of reconsultations for the same DRP confirms previous data suggesting the insufficient resolution of the primary cause as the main driver of early returns. In particular, the recurrence of gastrointestinal bleeding and epileptic seizures points to the need to optimize the dosing of anticoagulants and antiepileptics, as well as to reinforce education on warning signs [23,24]. Despite the expected increase in the 90-day reconsultation rate compared to the 30-day rate, the patient profile and the proportion of reconsultations due to new DRP episodes remained similar in both cases. Notably, the apparent predominance of “Other DRP” reflects the aggregation of multiple very low-frequency categories (each <3 cases); this composite group should be interpreted cautiously and does not support mechanism- or drug-specific inference.

Mechanistically, recurrent gastrointestinal bleeding and seizure revisits were often consistent with dosing issues, non-adherence, or underlying disease progression. In our setting, patients aged ≥80 years are frequently managed in geriatric-focused units and often have greater caregiver involvement, which might attenuate recurrence risk in this subgroup. Conversely, patients <80 years who lack such supports may warrant more targeted post-discharge follow-up: arrange a primary-care or community-pharmacist review within 7 days, offer simple adherence supports (blister packs/pill organizers/SMS reminders) for those living alone or with low health literacy, implement risk flags for high-recurrence regimens (antithrombotics, antiepileptics, diuretics, and hyponatraemia-inducing drugs), and document a brief social-support screen with a named contact.

Our study excluded index admissions ending in death and, due to the retrospective design and lack of linkage to a mortality registry, we could not systematically ascertain deaths occurring within 30 or 90 days among the screened cohort (including those excluded for prolonged hospitalization). Consequently, early ED revisit rates may be underestimated, and the apparently lower risk among patients aged ≥80 years should be interpreted cautiously, because differential mortality may have removed the frailest individuals from the population at risk (competing risk/survivorship bias). Future evaluations will incorporate registry linkage to quantify this bias prospectively.

Attribution limits: Because “Medication Code” is a multifaceted bundle (medication optimization, patient education, follow-up call, and a structured hand-off), these observational data cannot isolate which components (if any) drive the observed patterns. The bundle’s multifaceted nature precludes attributing outcomes to specific elements; future evaluations should use component analyses or factorial designs to estimate each component’s marginal contribution.

This single-center, retrospective study has important limitations. Internal validity is threatened by selection and survivorship biases (the exclusion of in-hospital and post-discharge deaths; the exclusion of prolonged stays), information bias inherent to retrospective ascertainment and non-blinded adjudication, and residual confounding (e.g., frailty, function, and social support). External validity is limited to settings similar to our tertiary ED and regional health system. Accordingly, our findings are descriptive implementation results and should not be interpreted as evidence of effect or generalized uncritically.

Our findings substantiate the significance of multifactorial secondary prevention programs within emergency settings and highlight the imperative to refine post-discharge follow-up protocols for elderly individuals and those identified as being at a high risk of recurrence. Prospective studies that compare varying inter-level coordination strategies and incorporate digital home monitoring technologies would be instrumental in further mitigating DRP-related revisits.

In conclusion, the “Medication Code” initiative retains its efficacy within routine clinical practice, exhibiting reconsultation rates that are analogous to those observed in controlled trials. The unexpected finding of reduced risk among elderly patients raises new questions about the roles of care settings and caregiver involvement in DRP prevention—questions that merit exploration in future studies.

## Figures and Tables

**Table 1:** Baseline characteristics of patients. Abbreviations: IQR, interquartile range; SD, standard deviation.

	Total (n = 120)	Reconsultation DRP (n = 37)	Reconsultation Other Cause (n = 83)	*p*
Age (mean; SD)	78.6 (16.2)	72.0 (19.3)	81.4 (13.8)	0.003
Female	71 (59.2)	19 (51.3)	52 (62.6)	0.245
Comorbidities				
Diabetes	36 (30.0)	8 (21.6)	28 (33.7)	0.181
Hypertension	97 (80.8)	26 (70.3)	71 (85.5)	0.050
Dyslipidemia	65 (54.2)	18 (48.6)	47 (56.6)	0.418
Chronic heart failure	30 (25.0)	7 (18.9)	23 (27.7)	0.304
Atrial fibrillation	52 (43.3)	19 (51.3)	33 (39.7)	0.237
Ischemic cardiomyopathy	20 (16.7)	4 (10.8)	16 (19.3)	0.250
Malignant diseases	27 (22.5)	10 (27.0)	17 (20.5)	0.428
Chronic renal failure	32 (26.7)	9 (24.3)	23 (27.7)	0.698
Dementia (Global Deterioration Scale 2–7)	40 (33.3)	8 (21.6)	32 (38.6)	0.069
No. of drugs at admission median (IQR)	9 (5–13)	7.9 (5–11)	9.1 (5–13)	0.177
Destination at discharge				
Home	67 (55.9)	23 (62.1)	44 (53.0)	0.351
Nursing home	12 (10.0)	4 (10.8)	8 (9.6)	0.843
Long-term healthcare center	16 (13.3)	2 (5.4)	14 (16.9)	0.088
Hospitalization	25 (20.8)	8 (21.6)	17 (20.5)	0.887
Days to reconsultation median (SD)	24.6 (22.8)	22.5 (23.17)	25.6 (22.7)	0.499

**Table 2:** Univariable and multivariable associations with 30-day DRP-related ED reconsultation.

	Univariate (*p*-Value)	Multivariate OR (CI95%)	*p*-Value
Age ≥ 80 years	0.001	0.32 (0.13–0.79)	0.013
Sex	0.246		
Hypertension	0.054	0.48 (0.18–1.30)	0.153
Chronic heart failure	0.307		
Diabetes mellitus	0.185		
Chronic renal failure	0.699		
Dyslipidemia	0.419		
Atrial fibrillation	0.238		
Ischemic cardiomyopathy	0.257		
Malignant diseases	0.429		
>10 drugs	0.149		
Dementia (Global Deterioration Scale 2–7)	0.073	0.70 (0.26–1.92)	0.492

## Data Availability

The data are available upon request due to restrictions (privacy).

## Abbreviations

The following abbreviations are used in this manuscript:

DRP	Drug-related problem
ED	Emergency department
OR	Odds ratio
CI	Confidence interval
IQR	Interquartile range
SD	Standard deviation
CHF	Congestive heart failure
ATC	Anatomical therapeutic chemical (classification system)
HSCSP	Hospital de la Santa Creu i Sant Pau
AIS	Área Integral de Salut (Barcelona Dreta)
EFAD	Equip de Farmàcia Assistencial de Barcelona Dreta (regional clinical-pharmacy network)
